# Physiotherapy assessment of breathlessness and disordered patterns of breathing: Defining a consensus on terminology and assessment

**DOI:** 10.1177/14799731251315483

**Published:** 2025-03-14

**Authors:** Lizzie JF Grillo, Izzie Easton, Fiona M Schreuder, Adam Lewis, Chloe I Bloom, Nicholas S Hopkinson, Harriet Shannon, Anne-Marie Russell

**Affiliations:** 1 National Heart and Lung Institute (NHLI), 4615 Imperial College London, London, UK; 2 Guys and St Thomas’ NHS Foundation Trust, Royal Brompton Hospital, London, UK; 3 School of Sport, Rehabilitation and Exercise Sciences, 2591 University of Essex, Colchester, UK; 4 7757 Pulmonary Rehabilitation, Swansea Bay University Health Board, Swansea, UK; 5 School of Health Sciences, 7423 University of Southampton, Southampton, UK; 6 Physiotherapy, Institute of Child Health, University College London, London, UK; 7 School of Medicine and Health, 150183 University of Birmingham, Birmingham, UK; 8 School of Nursing and Midwifery, Birmingham Regional NHS ILD and Occupational Lung Disease Service, University Hospitals Birmingham NHS Foundation Trust, Birmingham, UK

**Keywords:** Breathlessness, physiotherapy, breathing pattern disorder, assessment, terminology

## Abstract

**Introduction:** Abnormal breathing patterns unexplained by pathophysiology are typically referred to using terms including chronic breathlessness syndrome or complex breathlessness. Often patients with these conditions are referred to physiotherapy for an assessment of this breathlessness, where some are diagnosed with breathing pattern disorder (BrPD) or dysfunctional breathing (DB). The condition seen in physiotherapy occurs in at least 10% of the general population, increasing to 29−40% with coexisting conditions. Inconsistency in the nomenclature and physiotherapy assessment reduces recognition of the condition and hinders development in this area. **Aims of the study:** To establish expert physiotherapists' consensus on terminology to describe this condition and provide guidance for its physiotherapy assessment. **Participants and methods:** The opinions and experiences of ten respiratory physiotherapists, nine other clinicians (doctors, nurses, and speech and language therapists), and five patients diagnosed with BrPD were explored in focus groups or interviews regarding the terminology used and assessment experience. A second separate purposive sample of clinical expert physiotherapists (*n* = 11) took part in a nominal group technique (NGT) process to build consensus on the following questions: Question 1: What is your preferred term for this condition? Question 2: What are the most important assessment components to be included in all assessments? **Results:** One focus group (*n* = 10) and 14 interviews were completed. Framework analysis of the data from focus groups and interviews was undertaken and these results were shared with the participants in the nominal group. Consensus (71%) for the term breathing pattern disorder (BrPD) was achieved and an assessment guide was created. **Conclusion:** With improved consistency in its description and assessment, the adoption of breathing pattern disorder may help to further develop clinical and research priorities in this area within physiotherapy services.

## Introduction

The diagnosis and management of persistent breathlessness is a clinical challenge. The term “chronic breathlessness syndrome,” introduced in 2017, refers to breathlessness that persists despite adequate treatment and leads to disability^1^ although the term adequate is not defined in this context. In 2023 the European Respiratory Journal published a monograph on “complex breathlessness” which was defined as breathlessness without a clear cause or disproportionate to known pathology. These terms acknowledge that there are symptomatic breathless patients with potentially suboptimal treatments.

People with these conditions are often referred to physiotherapy services for breathlessness management where physiotherapists complete a detailed assessment of breathing, including its pattern and functionality. The complexities of these presentations are rooted in multifaceted characteristics of breathlessness, including neural, biomechanical, biochemical, cardiorespiratory, and psychological interactions.^2^ Such factors can cause breathing that deviates from allostasis (respiratory or metabolic needs) when conscious or unconscious processes override autonomic control.^3^ In some cases, this causes decreased arterial partial pressure of carbon dioxide via hyperventilation^4^ often influenced by psychological factors like heightened breathing vigilance.^5^ Individuals have worse physical functioning scores, are more anxious, and have poor health-related quality of life.^6,7^ Multiple terms are used to describe such presentations including, breathing pattern disorder, dysfunctional breathing and hyperventilation.^8^

### Nomenclature

In a national survey (pre-COVID-19), a self-selecting group of 103 UK respiratory physiotherapists indicated their preferred terms for this condition, predominantly breathing pattern disorder (*n* = 43%) or dysfunctional breathing (*n* = 39%).^9^ They expressed frustrations with the heterogeneous nomenclature and stated a consensus on terminology was urgently needed. Patients, too, have expressed frustrations with some not understanding what the terminology means and others feeling discomfort with their ‘label’ The lack of consensus was felt to diminish the importance of this condition, leading to diagnostic confusion^9^ and hampering clinical and research progress.

#### Assessment

Several assessment tools and outcome measures exist and unsurprisingly there is substantial heterogeneity in their measurement properties and some elements essential to a comprehensive assessment are missing.^10^ Objective assessment tools, such as the Manual Assessment of Respiratory Motion (MARM) are complex to use and the others including measurement of breath-hold time lack sufficient evidence to support use.^4^ In clinical research, many studies rely on the Nijmegen Questionnaire (NQ),^11^ whereas, in clinical practice, a more comprehensive physiotherapy assessment is completed.^9,12^ Cardiopulmonary exercise testing (CPET) and Opto-Electronic Plethysmography (OEP) are two further objective assessments. In people with this condition, CPET has shown a chaotic response to exercise with erratic ventilation and an increased, fluctuating respiratory rate, with larger tidal volumes.^13,14^ In athletes, OEP has shown biphasic changes during inspiration caused by paradoxical movement of the thorax, thought to undermine breathing performance.^15^ These studies have importantly progressed our understanding of the mechanisms behind this type of breathlessness.

With growing recognition of this condition and a rise in physiotherapy referrals, there is now an urgent need to better characterise it and develop consistent nomenclature and assessment approaches.^9,16^ Physiotherapists are well placed to lead on defining consensus of terminology with a patient-centred lens. We aimed to achieve this by using robust consensus-building methodology to provide exploratory evidence for physiotherapists’ preferred nomenclature for this condition and the necessary components for physiotherapy assessments, aiming to provide a springboard to improve definitions in this complicated area.

## Methods

This study was conducted in two stages. Stage 1:In focus groups and one-to-one semi-structured interviews we explored thoughts and perspectives on terminology and assessment of this condition with respiratory physiotherapists, clinicians, and patients. These were used to inform the next stage of this research. Stage 2: using the nominal group technique (NGT) we aimed to achieve consensus on nomenclature and assessment. Because we were interested in the terminology, we were sensitive to our choice of language and consistently used the term ‘this condition’ so as not to influence it.

### Stage 1 – focus groups and semi-structured interviews

#### Participants/recruitment

Purposive sampling was used to recruit to the focus groups and interviews. The inclusion criteria for Group 1 were UK-based respiratory physiotherapists with two or more years of experience in this area. Group 2 included clinicians (physicians, allied health professionals [excluding physiotherapists], or nurses) with experience working with patients with this condition for over 2 years. Group 3 included patients assessed and treated for unexplained breathlessness within a physiotherapy service. This included patients with co-existing disease and no pathophysiological reason for breathlessness. Physiotherapists were recruited through the UK professional body for cardiorespiratory physiotherapists, the Association of Chartered Physiotherapists in Respiratory Care (ACPRC), by expression of interest. Using a snowball sampling method, physiotherapy participants were asked to suggest non-physiotherapy clinicians and patients who the study team could approach as potential participants for groups 2 and 3. Due to unforeseen scheduling issues, a pragmatic decision was made to complete groups 2 and 3 as individual semi-structured interviews, which enabled a deeper exploration of concepts. Recruitment aimed for 6–10 participants per group.^17^

#### Procedures

Focus groups led by LG, supported by IE and FS, used a semi-structured topic guide (Appendix 1) to discuss terminology and assessment. The development of the guide was informed by published qualitative studies^9^ working with our Public and Patient Involvement and Engagement (PPIE) group. It was used to guide both focus groups and interviews to maintain consistency between the interviewers. The aim was to gather a breadth of insight from physiotherapists, clinicians, and patients to ensure representation from all key stakeholders. The information gathered would be shared with the nominal group to inform direct discussions and consensus and to ensure the nominal group were offered a broad range of opinions of these areas.

#### Analysis

The focus group and interviews were conducted and recorded using an NHS Microsoft Teams licensed platform (©Microsoft 2024). Recordings were transcribed verbatim, verified for accuracy by LG, pseudonymized by LG and securely stored on a password-protected computer. LG became immersed in the data by reading and re-reading the scripts. Using the framework method, we took a combined approach to analysis, enabling themes to be developed both inductively from the accounts (experiences and views) of research participants and deductively from existing literature and interview topic guide.^18^ Having identified a thematic framework, codes were assigned to the themes and subthemes. The research team LG, FS, and IE met and created a matrix of themes/subthemes traceable to the transcripts and participants. A descriptive step was used to explore themes, categories, and typology, linking overarching excerpts of narratives back to the data. Themes and research participants were grouped into higher-level categories/typologies based on similarities and linkages mapped between them.

### Stage 2 – nominal group technique (NGT)

#### Participants/recruitment

An additional purposive sample of UK-based experienced respiratory physiotherapists were identified for the NGT, with support from ACPRC. The NGT is a consensus-building methodology derived from the aggregation of members’ views. The optimal number of participants in a group is 8–12 for reliability of the group view and manageability of discussions.^19^ We invited 11 national clinical experts with at least 5 years of experience treating patients with this condition to work towards a consensus.^20,21^

#### Procedures

The NGT is a structured method for group thinking that encourages contributions from all participants and facilitates quick agreement and consensus.^22^ The NGT was held within a university setting led by AMR, experienced in this approach, with the support of the research team (LG, IE, HS, and FS). Instructions for the NGT exercise were given to the group via a presentation by LG before the commencement of the exercise. Participants consented to the study and had the opportunity to ask questions (Appendix 2 for NGT process).

#### Question for consensus

##### Question 1: What is your preferred term for this condition?

Each member of the group was given a list of terms used to describe this condition (Appendix 3-list of terms). The first list was informed by a review of search strategies from previous qualitative research and a systematic review^9,23^ (Appendix 4- search strategy). Participants were asked to consider terms independently for 10 minutes without discussion and rank them in order of preference. After this, a discussion was facilitated by the research team about the terms and their choices. The project lead (LG) provided a summary of the results from the focus groups and interviews completed in stage 1, to provide a broad reflection on the topics for consensus and to use alongside the NGT group’s own knowledge and experience. The same list of terms was then given to each participant with additional terms added from the focus groups and discussions. The group was asked to re-rank the terms (Appendix 3-list of terms). These results were shared with the group, and further discussion was facilitated. The revised list was presented, agreed terms were re-ranked, and consensus was defined as having been met when >70% agreement was reached.^24^

##### Question 2: What are the most important assessment components to be included in all assessments (subjective assessments, objective assessments, outcome measures)?

Participants were provided with a list of assessment components (Appendix 5), which included approaches described in published qualitative research^9^. The group was asked to categorise the approaches as 'core' or 'optional,' and invited to suggest additional components. ‘Core components’ were those deemed necessary for all assessments and ‘optional components’ those that may be used by more experienced clinicians or in specific circumstances. Facilitated discussions ensued, followed by a summary of findings from stage 1 (focus groups and interviews). A revised list was generated (Appendix 5), incorporating new components discussed in the first stage. Further discussions were facilitated until a broad consensus was reached. These discussions informed the development of an assessment guidance document, which was reviewed electronically by the group. Content Validity Index (CVI)^25^ was utilised to assess the guide’s validity. The CVI was calculated by dividing the number of experts endorsing that section by the number of experts (Appendix 6). Each part of the guide was scored out of four, and a score of more than three indicated endorsement by the experts.

### Analysis

NGT recordings were transcribed verbatim and reviewed to confirm the accuracy of the consensus discussions and rankings. These data were used alongside the rating and ranking outcomes from both the questions within the NGT. A rigorous interpretative approach to the analysis of the qualitative data was maintained throughout between researchers LG, IE, FS, and AMR. The research team are experienced in qualitative methods (LG/IE/AMR). Ethical approval for this study was received from the Health Research Authority Research Ethics Committee (REC) number 23/WA/0095 IRAS study ID 315897.

## Results

### Stage 1

One focus group (*n* = 10) and 14 interviews were completed. See Table 1 for participant characteristics. Code descriptions summarising the focus groups and examples of quotes are shown in Table 2 with further detail of the framework matrix is shown in Appendix 7.

**Table 1.** Description of participants.

Type of group	Gender	Profession (speciality) *experience*
Focus group: group 1 physiotherapists *n* = 10	All female	All UK physiotherapist B7 or equivalent (adult chronic lung disease)
		5–16 years clinical experience
Interviews	3 male: 6 female	1: Consultant respiratory medicine (asthma)
Non-physiotherapy clinicians *n* = 9		2: Consultant respiratory medicine (asthma)
		3: Specialist registrar (current asthma)
		4: Clinical lecturer respiratory medicine
		5: Clinical nurse specialist (asthma)
		6: Speech and language therapist (ENT and voice)
		7: Clinical psychologist (adult lung disease)
		8: Speech and language therapist (adult respiratory medicine)
		9: Consultant (respiratory medicine)
Interviews	4 females: 1 male	10: Participant with BrPD and COPD
Patients *n* = 5		11: Participant with BrPD and asthma
		12: Participant with BrPD
		13: Participant with BrPD asthma
		14: Participant with BrPD
Nominal group *n* = 11 (10+ years clinical experience)	8 females: 3 male	1 × Musculoskeletal and respiratory specialist
		8 adult chronic lung disease specialists
		2 clinical academic physiotherapists

**Table 2.** Description of themes and examples of quotes from participants.

Code	Description
Terminology	
Terms used for the condition	The different terms that clinicians and patients either use themselves or have heard being used to describe this condition
	Patient
	*‘There wasn’t an exact term, it was, I gotta be honest with you, I can’t remember exactly what they called it’*
	Physio
	*‘I think referral wise, we often get dysfunctional breathing written on a referral rather than breathing pattern disorder…..or disordered breathing..., but I think we tend to use breathing pattern disorder in our department’*
	Clinician
	*‘I think the top two end up being breathing pattern disorder or dysfunctional breathing pattern, I think those are the probably the most frequent ones that I come across and that I use’*
Strengths of a label	The positive impact of having a label for this condition, including the strengths of terminology
	Physio
	*‘It gives patients a label and gives some credibility to the diagnosis’*
	Clinician
	*‘Giving a label gives a pivot point to educate the patient about the problem they have’*
	Patient
	*‘I think having a label can sometimes help when I have to explain it (the symptoms) to a GP’*
Weaknesses of a label	The negative impacts/pitfalls of having a label for this condition, including the weaknesses of specific terms
	Physio
	*‘I think not for me as a professional, but for the patient when they’ve come across several different health professionals and they’ve got lots of different terminologies, it can be quite confusing for them’*
	Clinician
	*‘They might have been told that they’ve got breathing pattern, dysfunctional breathing pattern disorder or whatever term it is. But actually it hasn’t been explained to them what it is’*
	Patient
	*‘It can be difficult when you look it up online, there are lots of different descriptions and terms, some seem quite negative’*
Label and diagnosis	The value of using the label to diagnose this condition
	Physio
	*‘I * *t* *hink being diagnosed with a disorder or a dysfunction, especially when there’s no physiological test. That is very can be worrying for patients definitely'*
	Clinician
	*‘We have a responsibility to be clearer about it (the diagnosis/terms)’*
	Patient
	*‘Different clinicians often explain it in different ways which is confusing’*
BrPD and pathology	The specific issues that arise when this condition presents alongside pathophysiology
	Physio
	*‘We’ve had issues where people have asked for it (the term) to be taken off their letters, * *b* *ecause they’re scared that they won’t be taken seriously. So I guess, where does it become a breathing pattern disorder? * *P* *articularly when it’s overlapping with something else? It gets a bit complicated then’*
	Clinician
	*‘Often patients with both are much more complicated to treat and can take up more of your time’*
	Patient
	*‘It worries me that doctors don’t always take me as seriously when they see that I have breathing pattern problems. I worry they don’t take my asthma as seriously’*
Treatment considerations	Other information discussed about the treatment aspects of this condition
	Physio
	*‘Breathing pattern treatments are also helpful for those with general breathlessness’*
	Clinician
	*‘Continuity of physio/treatment is important for these patients’*
	Patient
	*‘Breathing exercises are hard when you have been breathing in a certain way for a long time’*
Assessment	
Subjective assessment	The components of subjective assessment included in an assessment of this type of breathlessness
	Physio
	*‘The patient story is so important in the subjective assessment’*
	Clinician
	*‘Understanding the referral story from primary care is really important'*
	*‘Differentiating from other types of breathlessness is important’*
Objective assessment	The objective components of assessment included in an assessment of this type of breathlessness
	Physio
	*‘The components of the BPAT are important in all assessments’*
	Clinician
	*‘A CPET test is as close to a GOLD standard assessment as we can get’*
Experience of assessment	The way an individual may experience an assessment of this type of breathlessness
	Physio
	*‘Subjective part of the assessment is the most important'*
	*‘Time will influence how many outcome measures you complete’*
	Clinician
	*‘Physiotherapists have the skills for a thorough assessment'*
	*‘Assessments are often long and not very consistent’*
	Patient
	*‘I didn’t realise until you explained which parts the assessments were verses the treatments’*

### Stage 2 nominal group

#### Question 1 Consensus on terminology

Discussions emphasised the need for a label to aid clinical consistency, acknowledging the interchangeable use of terms based on context. ‘Breathing pattern disorder’ and ‘dysfunctional breathing’ were deemed most clinically relevant, with the former favoured by 73%. Both terms were deemed to be accurate but there was a recognition that patients dislike ‘dysfunctional’. Summary of ranking of terms in rounds 1–3 are shown in Appendix 8.

#### Question 2 Consensus on assessment

**Figure 1.**
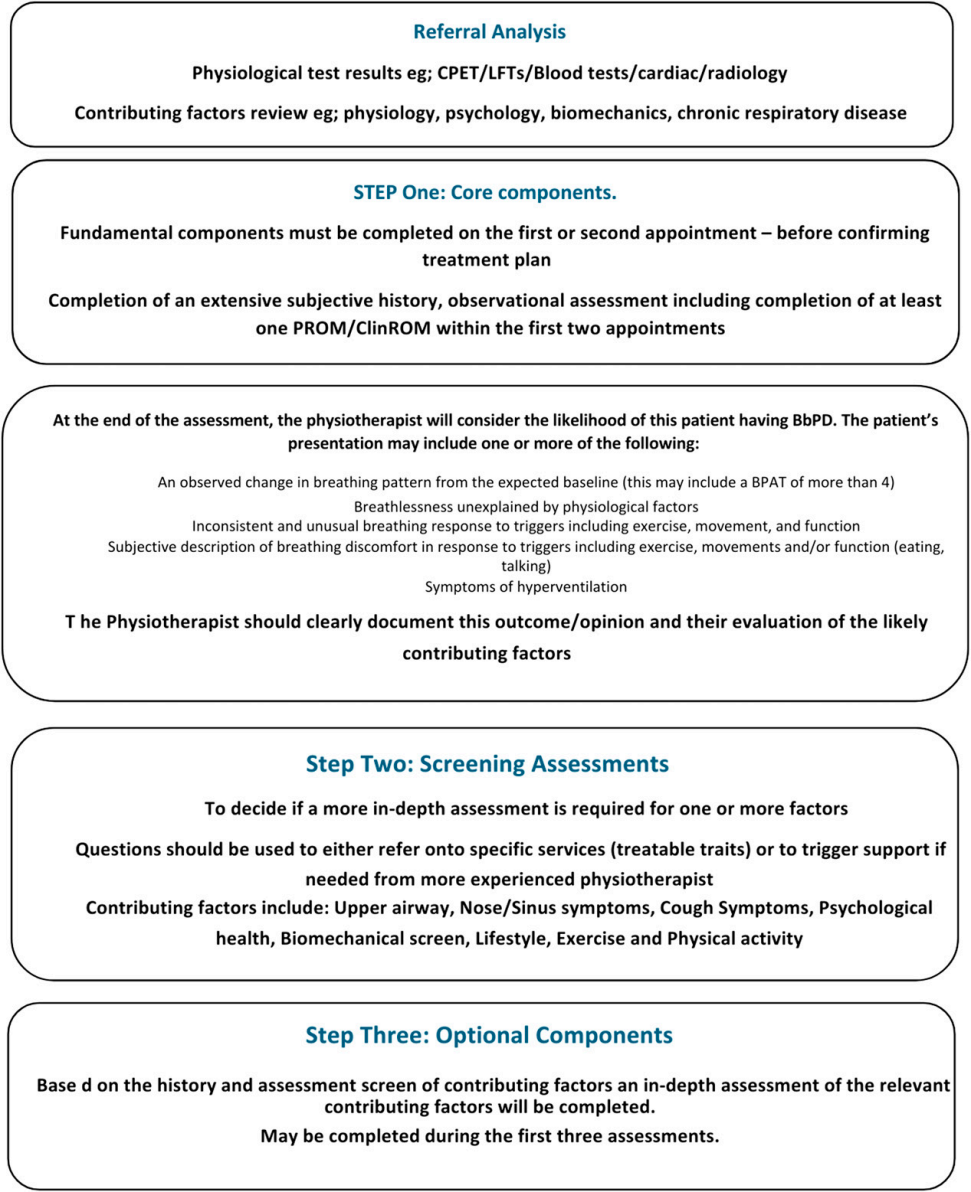
Summary of the assessment guide.

Discussion included the following areas: the definition of core assessments, minimum expectations of skill levels, and important components of assessment to enable a physiotherapist-led diagnosis of this condition. Participants expressed discomfort in omitting certain components entirely and sought to ensure that specific items were screened as part of the core assessment, though not necessarily fully assessed. This discomfort can be seen within the results of the two NG rounds where individuals found it difficult placing some components as core or optional (please see Appendix 9 for full results). Therefore, a guide was developed and informed by these results. The CVI Index on the first review (by the NG) of the document was 0.30 with 3 out of 11 clinicians endorsing the guide and, after edits, it increased to 0.8 with 9 out of 11 clinicians endorsing the guide. The nominal group was encouraged to provide comments at each review (Appendix 10, comments on the assessment guide drafts), comments were detailed including changes to wording, edits to the detail of certain components and consistency of terminology to improve overall clarity. A review of transcribed discussions afforded another level of quality and consistency checking. A summary copy of the guide is shown in Figure 1 (the full guide can be accessed online).

## Discussion

This is the first study to develop a consensus with expert physiotherapists’ preferred terminology emerging as breathing pattern disorder. Further discussions determined the acronym BrPD, includes a lowercase ‘r’ (not to be spoken), providing differentiation from other similar acronyms, for example, borderline personality disorder. This consensus was reached by a group of highly specialised expert physiotherapists and reflects terminology that can be adopted by physiotherapy services. This research also adds to the literature on assessment and provides a clear and usable guide for the assessment of BPrD within physiotherapy services. Importantly, we included insights from patients who are often under-represented in BrPD research.

### Significance of findings: Nomenclature

Previous research has suggested a consensus term would be important to clinicians and necessary to improve recognition of this condition.^9,26^ By using BrPD as the consistent term, physiotherapists can provide patients with a clear, dependable term for their symptoms, whilst physiotherapy services can align with the same terminology. This may provide a platform to encourage others to unify terminology. Consistent terminology may confer clinical credibility especially for those who feel their condition is under-recognised or dismissed. It also offers a starting point to enable systematic analysis, leading to a more rigorous synthesis of the literature and strengthening research, BrPD is not yet classified in the UK, lacking a Health Resource Group (HRG) code to reflect the increasing physiotherapy interventions. HRG codes group patient activities for payment by results through the use of procedure and diagnosis codes. The absence of a code may indicate poor recognition and missed clinical care opportunities. This consensus offers information to apply a usable code for BrPD, aiding in developing management pathways and better understanding treatment needs^27. ^An application (with support from the ACPRC and British Thoracic Society) requesting an HRG code for BrPD has been submitted.

Patient preference for terminology were an important component to consider and “dysfunctional” was unpopular among patients participating in this study. Focus group discussions indicated that patients felt that this term implied blame and diminished the importance of their symptoms and lived experiences. Many physiotherapists and clinicians suggested the term dysfunctional can make individuals feel they are doing something wrong, due in part to the dualism it fosters, (i.e. psychological explanation for symptoms is assumed) causing patient distress due to a lack of validation for the physical symptoms they are experiencing.

### Significance of findings: Assessment

The assessment guide has utility in supporting high-quality, repeatable clinical assessments. This creates a benchmark of what should be expected from a physiotherapy assessment and provides a credible foundation for further validation including physiotherapy education, clinical skill development, and service evaluation. The assessment guide builds upon previous research^9^ accounting for the knowledge and experience of the assessor. The guide is underpinned by detailed discussion and consensus between experts, including experienced clinicians with a range of expertise, representation from clinical expert groups and academics with experience in undergraduate and postgraduate training, and importantly informed by patients.

Further work informed by this assessment guide may provide the opportunity to develop the minimum skill level for physiotherapists undertaking BrPD assessments, as well as demonstrating the extensive assessment skills required with more experience.

This study provides valuable insights into the role of breathing pattern disorders (BrPD) and helps to highlight their importance within the broader context of breathlessness pathways. Assessing BrPD may be important in all cases of breathlessness including patients experiencing chronic or complex breathlessness who remain symptomatic despite optimised treatments.^28^ Identifying patients with BrPD is a clinical priority, as they may benefit from therapeutic interventions aimed at improving breathing patterns and overall function. The findings emphasise the importance of incorporating specialised expertise in breathing pattern assessment within services managing these patient populations. The assessment guide also recognises the importance of a holistic, biopsychosocial approach^29^ and includes additional elements essential in assessing breathing patterns i.e. psychological elements, upper airway, and performance. - all these factors are important to consider when developing the right therapeutic pathway for patients.^30^

### Methodological considerations

There are many methodologies suitable for gaining consensus including expert task force groups,^31^ consensus statements,^32^ and the Delphi technique.^33^ This research used the NGT, which represents a robust option for building consensus. It enables individuals to articulate and prioritise preferences independently in response to discussions and ensures the equal participation of all participants.^21^ Moreover, it provides prompt results for the research team and is more suited to discussion-based decision-making where it is important to generate a solution and evaluate the decision.^34^ It also capitalises on the expertise of the clinicians and enables them to engage in meaningful research due to its rapid format. Importantly, we included insights from patients with BrPD who are rarely represented in research.

The inclusion of focus groups and interviews before the nominal group was to ensure that the NG members had a balanced background of opinions to consider rather than only depending on their own views. The physiotherapy focus group attempted to widen the scope of inquiry established in the study published in 2023^9^ as well as ensure there had not been a change of opinion since COVID-19, where the interest in breathing pattern assessment as part of post-COVID assessment peaked. Additionally, the semi-structured interviews completed with other clinicians and patients added additional voices to these groups for the first time.

### Limitations

This study was conducted with clinicians and patients from the UK but may be generalisable given the insights gained. International discussion on nomenclature and assessment has been instigated and this methodology would be worth repeating more broadly in the international community.

We endeavoured to select a wide range of physiotherapists within the focus groups and nominal groups. Those interested in taking part may have had a preferred nomenclature. We acknowledge the lack of gender diversity within our physiotherapy focus groups (all female), however, we achieved more balance within the NG with 70% of participants being female, broadly in line with the female predominance of physiotherapists within respiratory services. We also acknowledge that other professional groups (including physiologists) were not included in this study based on the premise that BrPD is, in contemporary practice, mostly a physiotherapy diagnosis.

Whilst this study describes a preference for the term BrPD, robust studies evaluating possible mechanisms contributing to BrPD are lacking. There is a causality dilemma here – should the terminology be defined before or after the mechanistic studies? We hypothesise that mechanistic studies need the terminology to define study populations. We hope that by adopting consistent terminology within physiotherapy services, research will progress.

In conclusion, this paper presents a physiotherapy-specific consensus on the nomenclature and assessment of breathing pattern disorder (BrPD) within the UK, providing a starting point of a consistent framework for physiotherapy clinical practice. Furthermore, it underscores the broader importance of standardised terminology and assessment approaches in the field. A key contribution of this work is its recognition of the value of a clear label, offering patients validation of their symptoms and a better understanding of their condition when they are seen in physiotherapy services. BrPD, as a clinically acceptable and descriptive term, is not only beneficial for patient care but also holds the potential to advance both clinical practice and research in this area.

## Supplemental Material

**Supplemental Material -** Physiotherapy assessment of breathlessness and disordered patterns of breathing: Defining a consensus on terminology and assessment Supplemental Material for Physiotherapy assessment of breathlesness and disordered patterns of breathing: Defining a consensus on terminology and assessment by Lizzie JF Grillo, Izzie Easton, Fiona M Schreuder, Adam Lewis, Chloe I Bloom, Nicholas S Hopkinson, Harriet Shannon and Anne-Marie Russell in Chronic Respiratory Disease.
