# Supplementary material for: Physiotherapy assessment of breathlessness and disordered patterns of breathing: Defining a consensus on terminology and assessment
Source: Chron Respir Dis. 2025 Mar 14;22:14799731251315483. doi: 10.1177/14799731251315483 (PMC11909671; doi:10.1177/14799731251315483)
Supplement: Supplemental Material - Physiotherapy assessment of breathlessness and disordered patterns of breathing: Defining a consensus on terminology and assessment [file sj-pdf-1-crd-10.1177_14799731251315483.pdf]

Supplementary Information

Appendix 1 Interview/Focus Group Topic Guide

Question 1

Physiotherapists / Clinicians

a. What terms are used in your workplace to describe this condition ('this condition' refers to patients referred/ assessed as having unexplained breathlessness that cannot account for the severity or presence of breathlessness symptoms) and when do you use this term?
b. Do you have a preferred term for this condition?
c. Do patients with this condition all present the same?

For the patient interviews the questions were modified:

a. What term(s) did your clinicians use to describe your breathing condition? What other terms have you come across in your research
b. What do you think this condition should be called or shouldn't be called?

Question 2

Physiotherapists / Clinicians

What are the most important assessment components to be included in all assessments (objective assessments, subjective assessments, outcome measures)

For the patient interviews the questions were modified:
What assessments did your clinicians use in your assessment?
(Prompt if required: objective tests, questionnaires, exercise)

Appendix 2: Nominal Group process

The format for both questions presented to the nominal group was the following:

1) Presented with a list of terms/components

2) Asked to quietly and independently rank terms/components
3) Information handed to the research team and displayed as a group
4) First opportunity to discuss choices/results
5) Feedback from qualitative interviews
6) Presented with a second list of terms/components
7) Asked to quietly and independently rank terms/components
8) Second opportunity to discuss choices/changes? Results
9) Conclusion of question

Appendix 3: List of terms presented to nominal group

**List of Terms**	
**Round One**	**Round Two**
Dysfunctional breathing	Dysfunctional breathing
Thoracic dominant breathing	Thoracic dominant breathing
Over breathing	Over breathing
Breathing pattern disorder	Breathing pattern disorder
Air hunger	Air hunger
Unexplained breathlessness	Unexplained breathlessness
Breathing pattern dysfunction	Breathing pattern dysfunction
Deep breathing	Deep breathing
Apical breathing	Apical breathing
Sighing dyspnoea	Sighing dyspnoea
Thoraco-abdominal asynchrony	Thoraco-abdominal asynchrony
Forced abdominal expiration	Forced abdominal expiration
Hyperventilation	Hyperventilation
Hyperventilation syndrome	Hyperventilation syndrome
Psychogenic breathing	Psychogenic breathing
Disordered breathing	Disordered breathing
	Disrupted breathing
	Primary Breathing Pattern Disorder
	Secondary Breathing Pattern Disorder
	Thoraco-abdominal breathing
	Functional breathing
	Breathlessness

Appendix 4: Search Strategy informing List of Terms

1) dysfunction*" adj2 breath*
2) disorder* adj breath* NOT sleep
3) breathing adj pattern adj dysfunction
4) breathing adj pattern adj disorder
5) air hunger
6) irregular breathing
7) unexplained breath*
8) over* adj breath*
9) deep adj sighing
10) apical adj breathing
11) thoracic adj dominant adj breathing
12) thoraco-abdominal adj asynchrony
13) forced adj abdominal adj expiration
14) Subject heading exp Hyperventilation/ NOT epilepsy NOT sleep
15) Psychogenic breath*

Appendix 5 List of Components of Assessment presented to Nominal Group

Subjective

Pt description of symptoms
Pt's own awareness of breathing pattern
Triggers to symptoms
Recovery techniques/easing factors
Signs of air hunger (yawn, sigh, clear throat, tingling hands/feet)
Sleep (quality/duration)
Social History family/ work/ hobbies
Psychological History psychological illness/ stress & coping mechanisms
Nasal symptoms- blocked, runny, PND, altered sense of smell
Nasal symptoms- altered sense of smell
Exercise ability - frequency/intensity/time
Symptom description with exercise (Breathlessness/Cough/wheeze)
Physical activity levels
Voice/ Upper airway- voice changes, closure/discomfort in throat
Voice/Upper airway - closure/discomfort in throat
Cough type - dry/tickly/rattly/productive
Cough effectiveness

Results of other test/investigations

Objective
Observation of breathing- mouth/nose
observation of breathing- upper/lower chest
Observation of breathing - RR
Observation of breathing - air hunger
Observation of breathing - accessory muscle use
Observation of breathing - the rhythm of breathing
Observation of breathing- sounds on insp/exp
Observation of breathing - I:E ratio
Exercise/ functional symptoms (record method of assessment e.g. stairs)-changes to breathing pattern, WOB, Accessory muscles, cough
Voice- upper airway sounds, quality
Cough- nature, type, frequency; throat clearing
Postural assessment
ROM cervical spine
ROM thoracic spine
Core stability
Diaphragm Assessment- palpation
Diaphragm Assessment- movement
Core strength
*Rib cage movement**
*Observation of breathing in different positions**

Outcome Measures
Nijmegen Questionnaire
Breathing Pattern Assessment Tool (BPAT)
Short Evaluation Breathing Questionnaire (SEBQ)
Dyspnoea- 12
Optional PROM of psychological factors e.g., GAD-7, PHQ-9
Hospital Anxiety & Depression Scale
Breath Hold assessment
Manual Assessment of Respiratory Motion (MARM)
Ski Jump Assessment (BradCliff)
Exercise test e.g., 6MWT/CPET/SWT/Stairs
*BradCliff angle**
*Laryngeal hypersensitivity questionnaire**
*Cough questionnaire**
*Milstein Questionnaire**

*added for second round

Appendix 6: Content Validity Index

Assessment Document Feedback example question

This is a feedback form asking you for any comments on the assessment document sent as an attachment in the email. It asks you to kindly complete feedback as well as to score the relevance of each section. Thank you in advance for your help.

Please read through the first page of the assessment document titled 'Core components' and rate the relevance of the core components from 1 to 4; 1 being totally irrelevant and 4 being highly relevant.

1 2 3 4

Totally Irrelevant Highly relevant

Please use the next question to add additional comments for example if it is felt that the components are irrelevant/relevant or additional components need to be added/removed.

Appendix 7: Table of Framework Matrix from Focus groups and 1:1 interviews

	Physiotherapist	Clinician
NOMENCLATURE		
Terms/Labels used	Breathing Patten Disorder preference > Dysfunctional breathing<br>HV not useful<br>HV not useful<br>Disrupted breathing pattern<br>Disordered breathing pattern<br>Terms interchangeable	Breathing Pattern Disorder > Dysfunctional breathing<br>Disorder and dysfunction can be unpopular.<br>Hyperventilation if there is evidence.<br>Disordered breathing<br>Over Breathing<br>Unexplained breathlessness
Strengths of a Label	Multiple terms unhelpful<br>Consistency important<br>Gives patients reassurance.<br>Credibility to diagnosis	Consistency in label is important<br>Gives it credibility<br>Multiple labels confusing – needs consensus.<br>Overarching term important

	One label important	Label has an educational role
Weakness of a label	Language used important.<br>Patients can feel it's (the condition) their fault- with some labels<br>Patients can be upset by the label<br>Confusing abbreiviations	The word used can sometimes have negative impact<br>Label without explanation is unhelpful<br>Caution with using labels with some patients.
BPD and Physiology	More complex to Ax and Rx<br>BrPD and physiology occurs and is important<br>Caution with labelling when disease is present<br>Label in severe disease not always relevant.<br>Breathing pattern treatments for breathlessness without diagnosis being important	BrPD and physiology important<br>Much harder to assess and treat<br>Pathology and BrPD often have more healthcare usage<br>Incorrect label can be harmful<br>Important to work out driver for symptoms
Label and diagnosis	Diagnosis is challenging<br>Diagnosis of exclusion<br>Explanation of term alongside label<br>Physio best positioned to diagnose<br>Sometimes label less important that the treatment<br>Accuracy is important<br>Drivers for diagnosis are important	We have a responsibility to be clearer about it<br>SNOMED code would be helpful (criteria/consistency/track the condition)<br>Diagnosis with physiotherapy or skilled clinicians<br>Over or even under diagnosis may be occurring.<br>CPET often valuable for diagnosis<br>Diagnosis of exclusion
Treatment Factors	BrPD treatments are helpful with people that are breathless	BrPD treatments may help those with other conditions.<br>Continuity is important for patients with BrPD
ASSESSMENT COMPONENTS		
Subjective Ax	Patients lead the subjective<br>Subjective- triggers/aggs/eases<br>Subjective- when it started<br>Subjective description of episodes from the patient<br>Subjective description of Asthma v BPD<br>Check the referral for information<br>Medications- respiratory medication<br>PMH<br>Social history<br>Lifestyle – caffeine/contributing factors<br>-How long does it last<br>Chronic pain<br>Psychological history<br>Past trauma<br>Depression/stress<br>Expectations<br>Upper airway screening questions	Subjective history is key<br>Evidence from primary care is important<br>Gradual versus sudden (asthma differentiation)<br>Typical exacerbation description from the patient<br>Upper airway screen<br>Pain<br>Onset and nature of symptoms important<br>Unexpected or disproportionate symptoms<br>Evidence from primary care important

	Upper Airway Assessment in full	
Objective Ax	Covert assessment<br>Assessment of exertion/function<br>Physical exam<br>RR<br>Oxygen saturations<br>Volume<br>Rhythm and regularity of breathing<br>Auscultation<br>Nose breathing<br>Posture<br>Breathing in different positions<br>Ax in sitting and then in supine<br>*Cervical and Thoracic spine assessment*<br>*Rib cage movement*	Visual Assessment important<br>Mouth breathing v nose breathing<br>Exertional assessment important
Outcome Measures	BPAT++<br>D12<br>NQ<br>SEBQ<br>Breath-hold	CPET useful<br>Must check LFTs<br>BPAR useful<br>D-12<br>Questionnaires if used meaningfully.
Experience of Assessment	Subjective insightful<br>Long time required for assessment<br>Time will impact the choice of assessments<br>Subjective patient report important<br>Core Ax will change with experience<br>More depth of experience<br>NQ not always used<br>Breath Hold is often used<br>Upper airway Ax dependent on experience<br>An upper airway screen may prompt the patient or clinician to see any connection	Physiotherapists have the skills for the assessment<br>Results of assessment need to be concise<br>Conclusion of drivers to conditions important<br>Physiotherapy Assessment is not standardised which is difficult<br>The more objective tests the better<br>Subjective narrative from the patient is key<br>Mind and body important

Appendix 8: Summary of ranking terms

Ranking of terminology from Nominal Group Technique

	Number of participants ranking in top 3		Number of participants ranking as number 1		Final Vote
List of Terms	**Round 1**	**Round 2**	**Round 1**	**Round 2**	**Round 3**
Dysfunctional breathing[#]	10	6	2	0	3
Thoracic dominant breathing	1	0	0	0	-
Over breathing	1	0	0	0	-

Breathing pattern disorder#	***11***	***11***	***5***	***11***	***8 (73%)***
Air hunger	0	0	0	0	-
Unexplained breathlessness	0	0	0	0	-
Breathing pattern dysfunction	8	11	4	1	-
Deep breathing	0	0	0	0	-
Apical breathing	0	1	0	0	-
Sighing dyspnoea	0	0	0	0	-
Thoraco-abdominal asynchrony	0	0	0	0	-
Forced abdominal expiration	0	0	0	0	-
Hyperventilation	0	0	0	0	-
Hyperventilation syndrome	0	0	0	0	-
Psychogenic breathing	0	0	0	0	-
Disordered breathing#	3	4	0	0	-
Disrupted breathing+		1		0	-
Primary Breathing Pattern Disorder+		1		1=	-
Secondary Breathing Pattern Disorder+		0		1=	-
Thoraco-abdominal breathing+		0		0	-
Functional breathing+		0		0	-
Breathlessness+		0		0	-

+Round 2 terms, # Round 3 terms

Appendix 9: Summary of core and optional components defined by the NGT

Assessment components rated as core or optional from the nominal group technique

	Round 1		Round 2	
	Core components	Optional components	Core components	Optional components
Subjective				
Pt description of symptoms including patient's journey	11	0	11	0
Pt's own awareness of the br pattern	11	0	8	3
Triggers to symptoms	11	0	11	0
Recovery techniques/easing factors	11	0	10	1
Signs of air hunger	11	0	11	0
Social Hx- family/ work/ hobbies	11	0	10	1
Psychological Hx- psy illness, stress &	11	0	9	2

coping mech				
Exercise ability - frequency/intensity/time	11	0	8	2
Symptom description with exercise (SOB/Cough/wheeze)	11	0	8	2
Nasal symptoms- blocked, runny, PND	10	1	9	2
Physical activity levels	9	2	10	1
Cough type - dry/tickly/rattly/productive	9	2	5	3
Sleep (quality/duration)	9	2	5	4
Voice/ Upper airway- voice changes, closure/discomfort	8	3	8	2
Voice/Upper airway - closure	6	5	0	0
Cough effectiveness	6	5	2	4
Nasal symptoms- an altered sense of smell	6	5	0	0
Results of other tests/investigations			9	2
Observations				
Observation of breathing- mouth/nose	11	0	11	0
observation of breathing- upper/lower chest	11	0	11	0
Observation of breathing - RR	11	0	11	0
Observation of breathing - accessory muscle use	11	0	11	0
Observation of breathing - rhythm of breathing	11	0	11	0
Observation of breathing- sounds on insp/exp	11	0	11	0
Observation of breathing - I:E ratio	11	0	11	0
Observation of breathing - without patient awareness	11	0	10	1
Cough- nature, type, freq; throat clearing	11	0	4	5
Observation of breathing - air hunger	10	1	11	0
Postural assessment	10	1	9	2
Diaphragm Assessment- movement	8	3	7	4
Exercise/ functional symptoms (method of Ax e.g., stairs)	7	4	4	6
Voice- upper airway sounds, quality	5	6	2	7
ROM cervical spine	4	7	1	6
Diaphragm Assessment- palpation	4	7	5	4
ROM thoracic spine	3	8	2	6
Core stability	1	10	1	7
Core strength	1	10	1	6
Rib cage movement			7	4
Observation of breathing in different positions			5	5
Outcome measures				
Dyspnoea- 12	9	2	5	5
Nijmegen Questionnaire	8	3	4	5
Breath Hold assessment	7	0	5	2

Self-Evaluation Breathing Questionnaire (SEBQ)	5	6	3	4
Breath Hold assessment	7	0	5	2
BPAT	0	7	0	11
Optional PROM of psychology Ax e.g., GAD-7, PHQ-9	2	9	1	7
Hospital Anxiety & Depression Scale	0	11	0	6
Manual Assessment of Respiratory Motion	1	10	0	6
Ski Jump Assessment (Bradcliff)	2	9	2	4
Exercise test e.g., 6MWT/CPET/SWT/Stairs	2	9	2	7
Epworth sleep score	1	0	4	0
Bradcliff angle			1	4
Laryngeal hypersensitivity Questionnaire			1	5
Cough Questionnaire eg (LCQ)			1	9
Milstein Questionnaire			0	4

Green: 7 participants and above chose component, Yellow: 5-6 chose component, Orange: 4 or less chose component

Appendix 10: Comments on version 1 and version 2 of the Assessment of BrPD Guidance

Comments Section 1

ID	Name	Responses
1	anonymous	Do we need to include the frequency and recovery duration of symptoms in the subjective history?
2	anonymous	Cardiac - could be added as an example of a test. When triaging I would also be looking for some kind of acknowledgment from the referrer that Sx are in excess of stable or existing disease or no evidence of underlying disease/pathology contributing. I wouldn't include HR or SpO2 as fundamental core essentials at initial appointment.
3	anonymous	Agreed core components for completion at first assessment, or do we need to state before completing a treatment plan as often patients have a detailed history to tell limiting time to collect the core elements and complete everything on the 1st appointment.
4	anonymous	Overall, all the components are highly relevant with the following caveats: I would add to 'subjective': length of time that sx have been present plus whether they are getting better, same or worse = part of the patient's description. Re: observational: I don't usually have time to observe the patient's breathing during movement/exercise on

ID	Name	Responses
		the first assessment (because establishing a relationship with the patient and taking the subjective history predominates along with establishing baseline obs), although there will be objective measures of this if CPET data are available. (What is the difference between 'nose flow' in the observational column vs in the objective assessment? I think it fits better in the former unless nasal airflow is actually being measured). I always do the BPAT in the first session; nearly always do the D12 and very often do the NQ too, depending on the history and presentation.
5	anonymous	I think all are important, but I'm not sure sleep is a core component. The rest are essential for diagnosis and needed. I would also say that using the questionnaire ate the first appointment is a core as it opens a discussion about other sx we need to know about
6	anonymous	"lifestyle" - probably needs an expansion into work/activity/hobbies/Relationships - as any of these may be a significant driver to symptoms. Postures (in sitting and standing) would also be a useful initial observation.
7	anonymous	Physiological test results are relevant but not sure if contributing factors (physiology etc) are essential for the referral screen but would be relevant in the fundamental components to be explored in the initial assessment. Perhaps it needs to be in another box - an analysis of the first assessment? Are we saying that just observational assessment of breathing and movement only is essential? I feel this may need more clarification on how it might be done and is essential in the first appointment. I would do this with the type of patients that I see (athletes) but not sure how realistic it is for everyone. However, I am keen to include it though if we can as I think it's essential! sorry does that make sense? keen to pick this up within the essential and extended components of the BPD group study days once we have an agreement.
8	anonymous	Subjective assessment box: Suggest social history should be added and next to triggers put in brackets (initial and ongoing), history of the present condition (ie. history of onset of symptoms, progression so far, better/worse). Basic psychological history. Ask if the nose is blocked/ can they breathe through the nose or is this difficult? Sleep could be in contributing factor, page 2 section. Observational assessment box: Suggest adding muscle use and tension (ie. active expiration at rest, tense shoulders). Add to the observation of breathing during movement- observe breathing during 'trigger activity' (i.e.. movement/function/exercise/trigger activity). In objective assessment box: SpO2 a rest and during activity. Nose flow and I: E ratio (expiratory pause) is in both observational and objective assessment boxes- do we need both? Could Observational and objective assessment be merged into one box? BPAT should be in the core components section.
9	anonymous	All are highly relevant. Would agree that this 1st page captures the core components. Cannot think of anything missing here. Clearly set out.
10	anonymous	Does listing ‘expiratory pause’ under objective assessment imply it would be measured/timed? If so I wouldn’t include this in essential core components. I wouldn’t

ID	Name	Responses
		have deemed 'hands-on assessment' to be an essential objective measure in a first assessment either. I'd list CT scans alongside CPET/LFTs/blood tests in physiological test results.

Comments section 2

1	anonymous	None
2	anonymous	I don't think fatigue is as highly relevant as the other factors. I wouldn't describe sleep as a psychological factor, I would include it in lifestyle factors - which could be a separate box - sleep, diet, hydration, physical activity etc. Psychological would include, stress, anxiety, depression, PTSD, mental health diagnosis, social support/situation ...
3	anonymous	Important to recognise treatable traits and for precision medicine ensure they are seeing the correct professionals, but this wouldn't stop me from focussing on my scope of practice and offering a treatment plan.
4	anonymous	I agree that these should all be completed by the second assessment although I would normally have completed most of the UA, all the nasal, cough, fatigue, pain and psychological history by the end of the first assessment, especially if the issues are raised spontaneously by the patient. However, I would add that further support/guidance should be sought from not only a more experienced physio (if relevant) but also a doctor to address reflux, the need for nasal steroids, or perhaps a sleep study.
5	anonymous	i would suggest throat clearance possibly rather than throat sx. Sx in my experience are normally thought of as throat pain voice/loss and possibly not recurrent clearance- which isn't a cough
6	anonymous	In nasal assessment - writing "unable to nose breathe" would clarify that for nasal breathing to be "normal" the pt would need to be able to stipulate that it felt natural, i.e. that they were able to do it without conscious effort. see Zaghi 2020 DOI: 10.11648/j.ijo.20200601.13
7	anonymous	Does exercise need to be added as a contributing factor in its own box? Increased sensitivity to breathing also? e.g. underlying chronic illness or previous trauma/memory/panic
8	anonymous	Has 'nose blocked' and 'unable to nose breathe' already been covered in core components under nasal flow? I don't feel loss or change of smell is required in assessment as I feel it relates to whether they have nasal flow, which assessed separately. I feel the nasal and sinus assessment should be in the core components. I feel basic psychological history should be in the core assessment. Further in-depth psychological assessment could stay on page 2- contributing factors as this can take some time to assess and can't cover all of this in the first assessment. Should PND be under sleep section (if you mean paroxysmal nocturnal dyspnoea). Under the

		biomechanical screen box, I would add posture as a contributing factor. Objective assessment of AROM Cx and Tx (although equally could be in the third section-optional components). Drug history/ medication list and use could be contributory or optional sections.
9	anonymous	All are highly relevant. Some additional points I think for consistency e.g. related to some points where comments made on history e.g. infection / mental health/reflux history, however, no mention was made of chronic respiratory/cardiac disease history e.g. by cough which I think would be helpful for consistency. PMH is mentioned on 1st core components page but with these contributing factors it is important to mention all of the most common associations. Would also recommend adding pelvic floor history to my MSK biomechanical screen as I see this commonly i.e. complex/traumatic labor. Similarly with nose/sinus symptoms important to add any history of trauma/surgery/sinus disease here.
10	anonymous	Should include consideration of interactions with other health problems (e.g. Asthma, autonomic dysfunction, anxiety, long covid etc) I'd create a new section labeled 'lifestyle' in which I would include sleep. I wouldn't put this all under psychological history, as these may be contributing factors independent of psychological history. For example, they might include work patterns or social history. And should psychological history be called psychological well-being or health (includes factors that don't meet a formal psychological diagnosis and considers current psychological health independently of historical psychological health).

Comments section 3

1	anonymous	None
2	anonymous	I would include the Newcastle laryngeal hypersensitivity questionnaire for upper airway box 1. Cough could include Cough VAS - severity and frequency (quick and easy). Additional PROM could also include the B-HVQ
3	anonymous	Good, perhaps need to emphasise biomechanical review and assessment of core strength and quality of movements.
4	anonymous	I would add that assessment may need the skills of a doctor e.g. CLE, in addition to those of a more experienced practitioner. I agree that the other components listed are optional/could be completed by the 3rd session but would add depending on the initial presentation and success of treatment to date. I don't feel the Milstein assessment tool has been sufficiently 'validated'/used yet to include it in the BPD assessment. I don't use the breath hold test because I believe it is impossible to measure accurately although broadly it may be helpful to identify a possible problem I am not familiar with the Cottle manoeuvre or the Chalders Fatigue scale so can't comment on these
5	anonymous	Upper airway assessment use of Milstein measure and outcomes not helpful. I had to google then and I work with SLT. I think it might be better to list the components. Other outcome measures are fine. I can't see the Milstein as no access online for it .

		Could it be sent out? i think we do need to label the BPD questionnaires as BPD outcome measure not "additional PROM" While I would prefer they were not optional, I can see why you have placed them in optional
6	anonymous	Do you think that Breath Hold needs its own box? e,g around sensitivity /vigilance?
7	anonymous	Nasal flow is in both 'Core components' and 'optional components' I feel it should be in the core. Inspiratory and expiratory breath hold time could be additional outcomes. Not sure I fully understand "body position and reaction to stress" Is this about posture or how they position their body when stressed? Is this subjective or objective?
8	anonymous	As linked to question 4 - I think the history of symptoms would be better in contributing factors as fundamental rather than optional components eg upper airway / ENT history mentioned here, but I would mention on page 2.
9	anonymous	PEM (post exertional malaise) should be listed as PESE (post exertional symptom exacerbation) to encompass the full range of post exertional symptoms experienced. Fatigue outcome measures we commonly use is FAS (Fatigue Assessment Scale), rather than Chalders. The De Paul Questionnaire is also used by some to identify PESE (not sure if this is beyond scope of this project). Again, should this section include 'lifestyle/social'.

Appendix 11: Full assessment guide (please see separate upload/online file)
